# Analysis of the global trade network using exponential random graph models

**DOI:** 10.1007/s41109-022-00479-7

**Published:** 2022-06-11

**Authors:** Amin Setayesh, Zhivar Sourati Hassan Zadeh, Behnam Bahrak

**Affiliations:** University of Tehran, Tehran, Iran

**Keywords:** Complex networks, Trade networks, Exponential random graph models, Temporal exponential random graph models

## Abstract

The global trade network has significant importance in analyzing countries’ economic exchanges. Therefore, studying the global trade network and the factors influencing its structure is helpful for both economists and political decision makers. Putting these in mind, we try to analyze the global trade network from various viewpoints. We use the backbone filtering methods to construct a network of essential trades between countries. We analyze the structural, economic, geographical, political, and cultural factors and their effect on the global trade network using exponential random graph models. Additionally, we analyze the global trade network evolution using the separable temporal exponential random models. Our results show multiple structural, economic, geographical, and political factors affect the global trade network structure.

## Introduction

The global trade network has gotten significant attention in recent years due to its importance. In this network, countries are represented by their export/import relations and the amount and value of goods they transfer. Looking from different perspectives, this network can provide us with valuable information for political decisions and better insights into what is going on in the world economy. On this ground, the global trade network can be an interesting subject of study for both economists and researchers in social networks. Many questions about the world economy can be answered by studying it:

- Do rich countries tend to have more export than poor countries?
- Do political issues make countries less preferable for others to be the destination of the goods being exported?
- Do factors other than political and economic ones influence the formation of the trade network?

All these questions and more may be answered by studying the trade network structure and investigating the latent features extracted from it. The network structure of the global trade network can be glossed over and the result would be just data containing information about the involving countries and the corresponding amount of export among them. However, with the advances in social network analysis, more importance can be put on the network’s structure when studying such data. Features of the global trade network that pertain to the network’s structure, such as the triangular structures and other characteristics that originate from the network of the exchanges between countries, are pretty informative about the network processes and this can justify our endeavor to incorporate the structure of the network into our analysis. Observing the global trade network from different perspectives, various features and information other than the economic and political ones can be considered for analysis, which makes the study of this network an interdisciplinary area. An example of this would be including the data available on countries’ official languages, landlockedness, and country distances. So, to do an all-embracing analysis of the global trade network, one can add the features available on the countries and the network itself and take into account all the different aspects of this network. In this study, we use exponential random graph models (ERGMs), a family of models suitable to model the formation of dyads in relational data like network datasets. The main advantage of the ERGM method is its capability to incorporate different structural and non-structural features and even other networks into the analyses. We aim to model the global trade network from a social network perspective and incorporate different features available on countries and their relations in order to find the factors that affect the structure formation of this network.

Trade relation networks can be constructed and analyzed from different perspectives. One may study the trade network from the firm-level viewpoint; for example Chakraborty et al. (2021) and Krichene et al. (2019a, 2019b) study trades taking into account firms and their trade relations. Another approach is considering the bipartite network of trade exchanges. Chakraborty et al. (2019) used this approach and analyzed the bipartite network of banks and firms using the Bernoulli and the two-star model. Alternatively, we can perform a product-level analysis of the trade network; one example is Cingolani et al. (2017), which analyzes the trade flows in different industrial sectors. Our study analyzes and constructs the country-level trade network.

Multiple studies have been conducted on the global country-level trade network, each focusing on a different aspect. Most of the existing work look at the trade network from a non-structural perspective (Anderson and van Wincoop 2003; Helpman et al. 2007; de Soyres and Gaillard 2019), and do not consider the structural features of the network. As a step to take into account the structure of the global trade network (Bhattacharya et al. 2008), focused on the characteristics of the network analogous to the study that Benedictis et al. (2014) performed investigating the centrality metrics and the distribution of the statistics extracted from various features in the network. Also, Sajedianfard et al. (2021) worked on reconstructing missing edges in the network and analyzed the centrality and degree distribution of the constructed data.

As the next step to delve deeper into understanding the underlying formation process of the global trade network, modeling the formation of the network is done in different ways. Exploiting gravity models and other extensions of this family of models, De Benedictis and Tajoli (2011), Fagiolo (2010), and Dueñas and Fagiolo (2013) tried to model the global trade network. They incorporated the magnitude of the exports between countries and, focusing on gross domestic product (GDP), added this feature to their model too. Fagiolo (2010) built a residual trade network after applying the gravity equations and then compared the residual and original networks. Dueñas and Fagiolo (2013) predicted the weighted properties of the global trade network using gravity models.

Talking about the structure of the network as an integral part of our study, Almog et al. (2015) attempted to take into account the structural properties of the global trade network. They created a model based on countries’ GDP and predicted the weighted structure of the global trade network. However, less attention has been given to simultaneous considering of various features at both local and global levels, which can be done utilizing the power of the exponential family of random graphs. ERGMs are a rich family of random graph models used pervasively to model the formation of complex networks coming from various domains. The network of migration flows, and the factors involved in the formation of that network were modeled by Windzio (2018) using ERGMs. Additionally, the authors used temporal ERGMs to investigate the migration network evolution over time. They found that social network analysis, and ERGMs in particular, can effectively capture social processes’ embeddedness. Additionally, Gutiérrez-Moya et al. (2020) analyzed the global wheat network using ERGM approaches and found multiple features, including reciprocity, GDP, and country’s surface affecting the formation of wheat trades. The evolution of the global trade network is also analyzed in Cepeda-López et al. (2019), and Fagiolo et al. (2009). Also, taking into consideration the international trade network through time Abbate et al. (2012) analyzed the structures of the network. To the best of our knowledge, ERGM as a tool used to model the formation of the global trade network with simultaneous including of various structural, political, geographical, and economic features has not yet been done until now.

Overall, our contributions are primarily associated with finding and exploring the characteristics of the global trade network from different aspects. The trade relations are analyzed from temporal and static viewpoints in the study. Our results show that GDP, being landlocked, diplomatic exchanges, and distances are of more significance to the static trade relations between countries when considering all the various features. Additionally, structural features, GDP, inflation, being landlocked, and official languages are significant in the temporal analysis of trade relationships. Broadly speaking, the results of our study demonstrate the effective attributes alongside their importance in forming the global trade network in different levels of detail. Obtaining these factors and characteristics would allow the people working in various disciplines to interpret the network and its behavior either as a static or dynamic entity more accurately and based on the rich set of features that the network structure offers, in addition to the more country-specific features that one can incorporate modeling the network.

The rest of this study is organized as follows. The dataset and the methodology being used are described thoroughly in “Dataset and methods” section. “Results and discussion” section discusses the results of this study, and finally, future directions and conclusion are presented in “Conclusion” section.

## Dataset and methods

To examine trade network relations, we collected import and export trade data for the years 2011, 2013, 2015, and 2017. We enriched this network with the available information on each country and embedded various geographical, cultural, and political features. This part explains the methods and datasets used in our analysis. First, we present the trade network and the aggregation method used to create the directed trade network. Next, the network backbone extraction method used to keep the essential trades is described. Afterward, we present the other networks and features used for our research. After that, ERGM and STERGM, how we employed them, and the goodness of fit are described. Additionally, we provide an explanation of the gravity model of trade, which served as a baseline for the study. We conclude with the explanation of the community detection method used in our study.

### Trade network

**Fig. 1:**
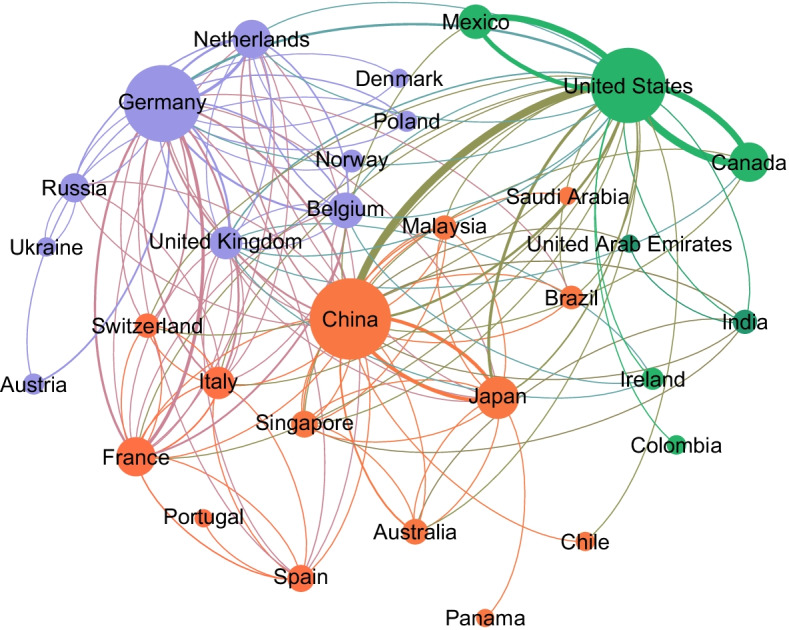
The trades network is depicted using a sample of all trades in 2011. Node sizes and edge thicknesses are proportional to degree and edge weight, respectively. The countries in the same community calculated using the Louvain algorithm are depicted using the same color

We got trade network data for the years 2011, 2013, 2015, and 2017 from the World Integrated Trade Solutions public website.^1^ These datasets contain details on a total of 241 countries and their weighted directed trade exchanges. To build the base network, we used the countries as the nodes and trades as edges alongside the amount of trade in terms of US dollars as the edge weights. The summary of trade network statistics for the year 2011 is shown in Table 1. A graph visualization of a sample of the global trade network is depicted in Fig. 1.

**Table 1:** Global trade network summary

Statistic	Value
Node count	241
Edge count	23,085
Graph density	0.39
Average degree	95.78
Average clustering coefficient	0.77
Average shortest path length	1.02

This dataset is a multi-edge network, which means multiple trades and their quantities are reported between countries. We calculated the total export and import between every two countries and used it as the weight of the edge connecting them. To create this new network, ReporterISO3, PartnerISO3, and Trade Value were used as the source, target, and edge weight, respectively. According to the World Integrated Trade Solutions definitions:

- The ReporterISO3 is the three-digit alphabetic country code for the importer country.
- The PartnerISO3 is also a three-digit alphabetic country code representing the exporter taking part in the trade.
- The Trade Value shows the amount of trade in thousand of US dollars.

### Network backbone extraction

We used the network backbone extraction method to develop an unweighted network containing just the essential trades in the network. Network backbone extraction is the process of reducing a graph to a more meaningful and compact representation. We applied the disparity filter (Serrano et al. 2009), which is a well-known network reduction algorithm, to the global trade network to only keep the essential trade exchanges. Other algorithms such as minimum spanning trees or thresholding methods can also be applied. However, we chose the backbone extraction method because it better perseveres the structure of the network and the essential edges. The disparity filter starts from the null hypothesis: the normalized weights which correspond to the edges of a certain node are produced by a random assignment from a uniform distribution. Next, defining a significance level and the probability density function, the filter keeps significant edges to act as the backbone of the graph. We used the significance level 0.2 in our study. We chose the significant level by an experiment in which we applied different significant levels and observed the count of the remaining edges. Serrano et al. (2009) proposes this method of progressively changing the significance level to focus on the more relevant edges. The probability density function is provided in Eq. 1 where *k* is the degree value.

1 $$\begin{aligned} \rho (x)dx = (k-1)(1-x)^{k-2}dx, \end{aligned}$$

We used the network resulting from the disparity filter as an unweighted network representing the backbone of the initial trade network.

### Features

#### Nodal features

We used multiple features in our analysis, gathered from the World Bank^2^ and the CEPII public datasets.^3^ Data on global development for various countries are available free and openly through the World Bank. Similarly, the CEPII website provides the GeoDist dataset, containing the country’s official language, details about the colonial history, and geographical variables, including continent, landlocked status, and area. The list of nodal features and their statistical properties for the year 2011 are provided in Tables 2 and 3.

**Table 2:** Summary statistics of numerical node features for the global trade network

Node feature	Count	Mean	STD	Min	Median	Max
GDP (Current US$)	261	2.29e+12	7.94e+12	3.87e+07	4.19e+10	7.34e+13
Inflation, consumer prices	202	6.01	5.80	− 0.40	4.62	53.22
GDP growth	259	3.60	5.71	− 62.07	4.03	21.67
Foreign direct investment, net inflows	200	1.18e+10	3.98e+10	− 6.00e+09	9.99e+08	3.32e+11
Agriculture, forestry, and fishing, value added (% of GDP)	197	11.10	11.23	0.03	7.44	54.59
Industry (including construction), value added (% of GDP)	197	27.38	15.03	4.16	24.67	80.00
Merchandise trade (% of GDP)	198	72.73	49.42	14.46	62.02	435.21
Net barter terms of trade index (2000 = 100)	201	127.50	56.07	49.61	104.89	447.02

**Table 3:** Summary statistics of categorical node features for the global trade network

Node feature	Count	Unique	Top frequency value	Top frequency
Is landlocked	208	2	False	170
Official language	208	64	English	60

#### Distance network

As an edge feature, we used the GeoDist distance network dataset (Mayer and Zignago 2011) in our study. This complete graph consists of 224 countries and 24,976 pairwise dyadic distances. A summary of this network is provided in Table 4. The GeoDist dataset provides two geographical distances: simple and weighted. The weighted distance is calculated using several principal cities in each country, and its primary usage is consistently computing internal and international distances. We did not include internal trades in our study, so we used the simple geographic distance. The simple distance is calculated using the great circle formula utilizing the latitudes and longitudes of the most important city in each country in terms of population. The nodes and edges in the distance network are defined as countries and distances, respectively.

Using the dataset, we include the distance between countries. This is compatible with the gravity trade model suggestion of relation of trade and distance between countries (Leibenstein 1966).

**Table 4:** Distance network summary

Statistic	Value
Node count	224
Edge count	24,976
Graph density	1
Average degree	223
Average clustering coefficient	1
Average shortest path length	1

#### Diplomatic exchange network

To consider the effect of political relations between countries, we used the Diplomatic Representation dataset (Moyer et al. 2016) from the PARDEE website^4^ which provides details about the country relations across time. We used this dataset to construct the diplomatic relations network between countries keeping countries as the nodes and directed edges representing the source country having an embassy in the target node. A summary of this network is provided in Table 5.

**Table 5:** Diplomatic exchange network summary

Statistic	Value
Node count	201
Edge count	8959
Graph density	0.22
Average degree	89.14
Average clustering coefficient	0.73
Average shortest path length	1.70

#### Colonial history network

We created a colonial history network using the dataset provided by Harvard Dataverse (Walter et al. 2019). This network consists of 107 directed colonization relations such that the directed edge from node A to node B shows country A has been colonized by country B. A summary of this network is provided in Table 6.

**Table 6:** Colonial history network summary

Statistic	Value
Node count	90
Edge count	107
Graph density	0.01
Average degree	2.37
Average shortest path length	0.01

### ERGM

ERGMs also known as p-star models (Wasserman and Pattison 1996) allow analyzing tie creation in networks. One fundamental assumption that makes ERGMs different from regression models is that network ties aren’t independent, and multiple processes can operate simultaneously. The independence assumption is not realistic in most real-world processes, and ERGMs provide more sensible models that capture the dependence between network ties. They describe the local selection forces that shape the global structure of a network (Hunter et al. 2008). ERGMs allow us to include and consider nodal features, structural configurations, and edge features.

The probability distribution used in ERGMs is provided in Eq. 2

2 $$\begin{aligned} Pr(Y = y) = \frac{exp(\theta ' g(y))}{k(\theta )}, \end{aligned}$$

where *Y* is the random graph variable, *y* is an instance of *Y*, $\theta$ is a vector of coefficients, and *g*(*y*) is a vector of graph statistics. $k(\theta )$ is the normalizing term and is defined in the Eq. 3.

3 $$\begin{aligned} k(\theta ) = \sum _{y'} exp(\theta ' g(y')), \end{aligned}$$

where the sum is taken over the whole sample space of allowable networks $y'$ (Hunter et al. 2008). By exploiting this model and fitting it to the network at hand, we’re trying to find a set of parameters for our model to which, if we feed a network similar to our observed network in certain features, it will assign a higher likelihood of observing.

We used the statnet package (Hunter et al. 2008) in order to fit our model using ERGMs. The model with dependant terms is fitted using the Markov Chain Monte Carlo algorithm to estimate $\theta$, maximizing the likelihood of the observed edges configurations.

We used the 2011 trades for this analysis because it was the completest dataset among all collected datasets. Also, because the countries differ in different feature networks, this analysis utilizes countries at the intersection of the global trade network, the diplomatic exchange network, and the distance network.

#### ERGM terms

As mentioned in the “ERGM” section, *g*(*y*) is a vector of graph statistics, and the frequency of a specific configuration defines these graph statistics. Various terms can be defined to determine the network statistics in the *g*(*y*) vector. The definitions of these terms are described below.

*Edges* This term is defined as the total number of edges in our graph. We can control our graph’s density using this term.

*Mutual* It is defined as the number of pairs *i* and *j* for which edges (*i*, *j*) and (*j*, *i*) both exist. This configuration is commonly known as reciprocity which means tending to exchange both ways.

*Triangle* It’s defined as the number of triangles in the network. For an undirected network, a triangle is defined by any set of (*i*, *j*), (*j*, *k*), (*k*, *i*) of three edges. For a directed network, a triangle is defined as any set of three edges (*i*, *j*) and (*j*, *k*) and either (*k*, *i*) or (*i*, *k*).

*Nodecov* It’s defined as the sum of $attr(i) + attr(j)$ for all edges (*i*, *j*) existing in the graph. The *attr* can be any quantitative nodal feature present in the graph. This configuration is useful for knowing how the sum of continuous node features affects tie formation.

*Absdiff* It’s defined as the sum of $(abs(attr[i]-attr[j]))^{pow}$ for all edges (*i*, *j*) existing in the graph; we used the first power in our analysis. Similar to the Nodecov term, *attr* can be any quantitative nodal feature. It is used as a homophily configuration for continuous variables. Homophily is the tendency for similar entities to be attracted to each other.

*Nodematch* It’s defined as the count of edges (*i*, *j*) for which $attr(i)==attr(j)$, where *attr* can be any categorical nodal feature. It captures homophily on categorical features.

*Edgecov* Other networks can be used as controlled network statistics using this term. It’s defined as the sum of edge features for each edge (*i*, *j*) present in the network.

### STERGM

Although ERGMs allow for analyzing tie creation in networks, they cannot include the effects of network evolution over time. In order to take into account the network evolution over time, Discrete Temporal ERGMs (DTERGMs) were introduced (Hanneke et al. 2018).

DTERGM is an exponential random graph model that defines the network at time *t* as a single draw from an ERGM conditional on the network at time $t-1$. The probability distribution for this model is formulated in Eq. 4.

4 $$\begin{aligned} Pr(Y^t = y^t | Y^{t-1} = y^{t-1}, \theta ) = \frac{exp(\eta (\theta ) g(y^t, y^{t-1}))}{c_{\eta ,g}(\theta , y^{t-1})} \end{aligned}$$

Where *c* is the normalizing constant, the $y^t$ term represents the network at time t, and the $\eta$ term shows a mapping from $\theta$ to natural parameters.

One disadvantage of DTERGMs is that they consider network formation and dissolution as a single process. However, processes and parameters which affect tie formation and dissolution are not the same. Considering this issue, Separable Temporal ERGMs (STERGMs) were introduced (Krivitsky and Handcock 2013).

STERGMs consider two different phases in network evolution: formation and dissolution. The formation process analyzes ties being created; the dissolution phase considers removed ties. This is a more realistic approach due to different processes in the formation and dissolution of ties in the network. In our work, we used STERGMs to analyze the global trade network’s evolution from 2011 until 2017.

### Goodness of fit

To determine whether our model is a good fit for the global trade network, we use the goodness of fit test proposed by Hunter et al. (2008). This approach generates sample networks. Then, the model configurations are calculated on both the observed network and the generated networks. Finally, the distribution of statistics in the samples are compared with the observed network. Multiple network statistics can be used. We included minimum geodesic distance, triad census, in degree, and out degree in our analysis. The statistics are explained below.

*Minimum Geodesic Distance* This statistic is a measure of the distance between two nodes. We calculate it as the number of edges in the shortest path between two nodes.

*Dyad-wise Shared Partners* We define this statistic as a function of *s*, where *s* is the number of shared partners. The statistic equals the number of edges in the network with *s* shared partners.

*Degree* The degree is calculated as the number of edges connected to a node. Similarly, in-degree and out-degree are calculated as incoming and outgoing edges count, respectively.

*Triad Census* It is defined as the number of subgraphs with three vertices, regardless the direction of the edges between them.

### Gravity model of trade

The gravity models are derived from Newton’s Law of Gravitation. These models are frequently used De Benedictis and Tajoli (2011), Fagiolo (2010) and Dueñas and Fagiolo (2013) to analyze trade networks by economists and are fundamentally different from ERGMs. The gravity model of international trade states that the volume of trade between two countries is proportional to their economic mass and a measure of their relative trade frictions (Baier and Standaert 2020). The naive gravity model is formulated as Eq. 5.

5 $$\begin{aligned} X_{ij} = GY_{i}^{\beta _1}Y_{j}^{\beta _2}dist_{ij}^{\beta _3}\epsilon _{ij}, \end{aligned}$$

Where $X_{ij}$ is bilateral trade between exporting country *i* and importing country *j*, $Y_{i}$ and $Y_{j}$ are the GDP in country *i*, *j* respectively and $dist_{ij}$ is the bilateral distance between country *i* and *j*. $\epsilon _{ij}$ is a log-normaly distributed error term (Baier and Standaert 2020). The countries’ economic mass is represented by their GDP, and the trade frictions are represented by country distances. The gravity model suggests that distance has a negative effect on trade exchanges. It also proposes the fact that counties’ economic size attracts them to trade with each other.

Our study used gravity models of trade to compare ERGM findings with the commonly used gravity model of trade estimations in economics.

### Community detection

We applied Louvain community detection (Blondel et al. 2008) to the global trade network. This algorithm is based on the idea of maximizing modularity. The modularity measures the community’s inner edges density compared to the other edges in the network. Louvain’s algorithm starts with small communities and iteratively merges them to find the communities with the maximum modularity. Without applying the backbone extraction method, the complete weighted network was used for community detection.

## Results and discussion

In this section, we provide the results of our analysis and their interpretations. First, we provide the ERGM and STERGM results and the significant features affecting the global trade network. Second, the results of the gravity model of trades are shown. Finally, we interpret the results of community detection using the colored world map.

### ERGM

We found various significant features that may affect trade network relations formation through the ERGM model analysis in the year 2011. These results are shown in Table 7. Taking into account structural characteristics, we observe positive reciprocity and transitivity effects. The positive reciprocity effect indicates that countries tend to trade in both directions rather than in one direction. This pattern indicates that if country A exports to country B, there is a high tendency for country B also to export goods to country A. The ERGM analysis on the wheat trades Gutiérrez-Moya et al. (2020) also found this reciprocity effect significant on the formation of the edges in the network. The transitivity effect shows an interesting triangle formation effect in the trade network. It suggests triangle patterns occur more in the trade network than in random networks. In other words, two countries that have trade relations with a third partner have a tendency to start doing trade together. These structural findings are in alignment with the structure of the global trade network. We also considered other structural features, but there were problems with convergence, so we did not include them.

From the non-structural features perspective, we found several significant features. The results suggest a positive effect of trade partners’ combined GDP in the trade network, which means the sum of GDP in both countries affects the formation of ties between countries. Moreover, absolute GDP differences have a positive effect, indicating interesting homophily effects regarding countries’ GDP. In other words, the closer GDP values between countries, the more likely it is that the countries form trade relations. The GDP findings align with previous works such as Fagiolo (2010), and Dueñas and Fagiolo (2013). Also, our gravity model results, provided in Table 9, suggest a significant effect of GDP on the global trade network. Also, ERGM-based studies such as Gutiérrez-Moya et al. (2020) found GDP has a significant effect on trades. No effect of inflation and GDP growth is shown in the results, which we expected. These results are in alignment with our gravity model results. However, the absolute difference in GDP growth is significant in the gravity model results. We assume this is due to the gravity model not including structural features that affect the trade network formation.

Considering the geographical features, the results indicate a significant effect of landlockedness on edge formations. landlockedness is defined as being enclosed by land and having no route to the sea. This finding can be explained by the fact that most trades are transported through the ocean. We also find that geographical distances between countries affect trade formations. It agrees with the gravity model of trades, suggesting that trade volumes and distance affect trades.

One surprising result was the negative effect of diplomatic relations on trade exchanges, while we expected a positive impact of diplomatic relations on edge formations. The negative impact can be explained by the heterogeneous effect of diplomatic relations on trade, that according to Hinz and Leromain (2020), can vary within pairs of countries.

We also find other economic features related to the percentage of GDP significant. These results are consistent with the gravity model results of the trade. Another interesting result is the “Net Barter Terms of Trade Index.” The “Net Barter Terms of Trade Index” is the ratio between the price of a country’s export goods and import goods. The results show a positive effect of the sum of the “Net Barter Terms of Trade Index” between countries on trade relations. However, we observe a negative effect of the absolute difference of the “Net Barter Terms of Trade Index.” In other words, the closer the countries are together regarding the “Net Barter Terms of Trade Index,” it adversely affects edge formation in the network. Net Barter Terms of Trade Index results are consistent with the gravity model of trades in both significance and positivity. We find no effect of colonial history relations on the trade formations. It is likely because the current country relations are independent of what colonization they have undergone. Today’s relations between countries are not based on former colonial relationships.

**Table 7:** ERGM estimates and standard errors alongside their significance on edge formation

Configuration	Estimate	Standard error	Significance
Edges	− 4.545	0.292	***
Mutual	0.212	0.067	**
Triangle	0.030	0.003	***
GDP (Current US$) nodecov	0.185	0.020	***
GDP (Current US$) absdiff	0.272	0.024	***
Inflation, consumer prices nodecov	− 0.040	0.022	.
Inflation, consumer prices absdiff	0.036	0.028	
GDP growth nodecov	− 0.010	0.017	
GDP growth absdiff	− 0.027	0.023	
Is landlocked nodematch	− 0.220	0.039	***
Official language nodematch	− 0.014	0.063	
Diplomatic exchange network edgecov	− 1.677	0.079	***
Agriculture, forestry, and fishing, value added (% of GDP) nodecov	− 0.052	0.022	*
Agriculture, forestry, and fishing, value added (% of GDP) absdiff	0.090	0.028	**
Industry (including construction), value added (% of GDP) nodecov	0.055	0.020	**
Industry (including construction), value added (% of GDP) absdiff	− 0.114	0.026	***
Merchandise trade (% of GDP) nodecov	0.046	0.017	**
Merchandise trade (% of GDP) absdiff	− 0.031	0.026	
Net barter terms of trade index (2000 = 100) nodecov	0.040	0.015	*
Net barter terms of trade index (2000 = 100) absdiff	− 0.090	0.024	***
Foreign direct investment, net inflows nodecov	− 1.474	0.199	***
Foreign direct investment, net inflows absdiff	1.352	0.200	***
Colonial History network edgecov	− 0.513	0.603	
Distance network edgecov	0.190	0.030	***

$$p<0.1$$^**.**^; $$p<0.05$$^*^; $$p<0.01$$^**^; $$p<0.001$$^***^

### STERGM

**Fig. 2:**
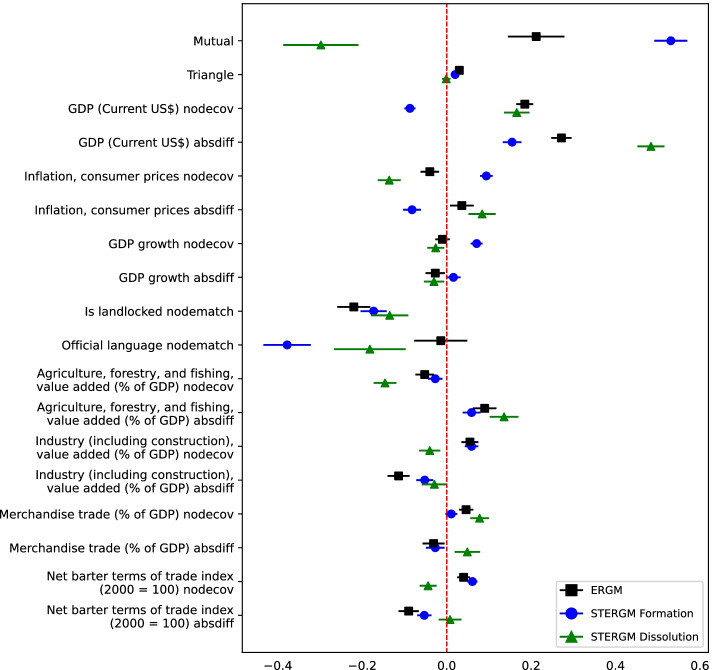
ERGM and STERGM Results depicted using the Dot-and-Whisker plot. ERGM, STERGM formation, and STERGM dissolution are shown for each feature

We analyzed the global trade network evolution using the STERGM method. The results are provided in Fig. 2 alongside the ERGM results. The STERGM formation and dissolution estimates are shown using the blue and green colors, respectively. Additionally, the formation and dissolution significant features of STERGM are shown together in Table 8.

Comparing formation and dissolution’s significant features show exciting insights. There is a significant effect of reciprocity on the formation and dissolution of edges through time. This means that mutual edges in the network affect the processes of forming and dissolving edges through the years. According to Fig. 2 reciprocity has different effects on the formation and dissolution of edges in the network. Edge formation is positively impacted by reciprocity. When country A exports to country B, it’s highly probable that the trade will also take place from country B to country A. However, reciprocity negatively affects dissolution. It highlights how similar configurations can affect the formation and dissolution of ties in a network differently. The reciprocity result shows that when mutual edges are formed, they prevent the dissolution of edges in the network. Another structural configuration included in the temporal network analysis was the occurrences of triangles. The results show a significant effect of triangles on the edge formation through time, but no such effect is seen on the dissolution of the edges in the global trade network. The estimates show a positive impact of triangles on forming the new edges in the network.

In terms of nonstructural features, similar to the ERGM results, the sum of GDP between countries and the absolute difference is significant in the STERGM results. One interesting difference between the ERGM and STERGM results is the effect of inflation on the trades. ERGM results show no significant impact of inflation, but STERGM results show that inflation significantly affects formation and dissolution processes in the network. The landlockedness STERGM results are also similar to the ERGM results. ERGM results show no significant correspondence between official language similarities and official language differences. In contrast, STERGM considers similarities of official languages to have a negative impact on the formation and dissolution of trade edges. The results were unexpected because we expected similarity in the official languages to indicate a tendency for trade. It might be the case that fewer countries face language barriers nowadays.

**Table 8:** STERGM features’ significances

Configuration	Formation significance	Dissolution significance
Edges	***	***
Mutual	***	***
Triangle	***	
GDP (Current US$) nodecov	***	***
GDP (Current US$) absdiff	***	***
Inflation, consumer prices nodecov	***	***
Inflation, consumer prices absdiff	***	**
GDP growth nodecov	***	
GDP growth absdiff		
Is landlocked nodematch	***	**
Official language nodematch	***	*
Agriculture, forestry, and fishing, value added (% of GDP) nodecov		***
Agriculture, forestry, and fishing, value added (% of GDP) absdiff	**	***
Industry (including construction), value added (% of GDP) nodecov	***	
Industry (including construction), value added (% of GDP) absdiff	*	
Merchandise trade (% of GDP) nodecov		***
Merchandise trade (% of GDP) absdiff		
Net barter terms of trade index (2000 = 100) nodecov	***	*
Net barter terms of trade index (2000 = 100) absdiff	**	
Foreign direct investment, net inflows nodecov	***	
Foreign direct investment, net inflows absdiff		

$$p<0.1$$^**.**^; $$p<0.05$$^*^; $$p<0.01$$^**^; $$p<0.001$$^***^

### Goodness of fit

**Fig. 3:**
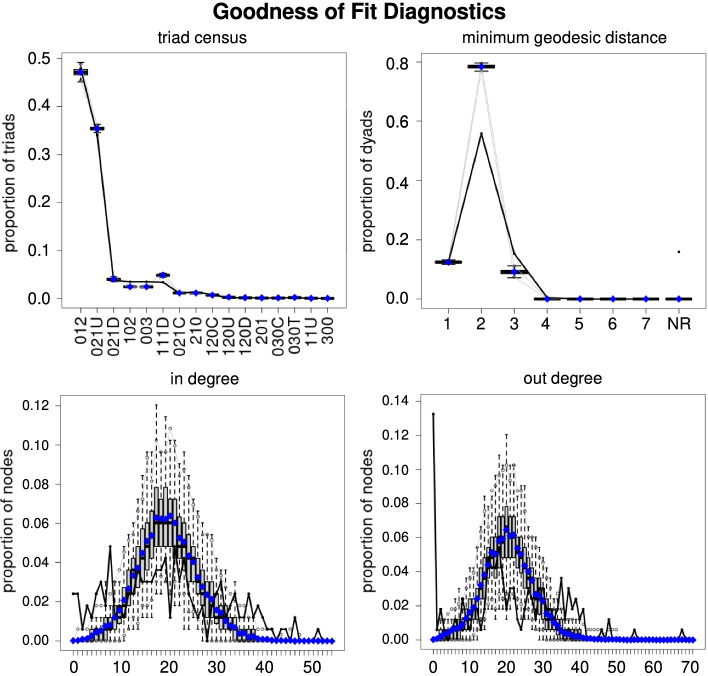
The Goodness of Fit results in the simulated networks. The minimum geodesic distance, triad census, in degree, and out degree are captured. The blue points in the plot represent the mean of statistics in the simulated networks. The black line shows the observed statistics in the actual network

The results of the goodness of fit analysis are provided in Fig. 3. Results indicate that the model accurately captures the minimum geodesic distances and triad census, as both simulated and observed results are similar. The in degree and out degree of countries are also shown in Fig. 3. Compared to minimum geodesic distances and triad censuses, these statistics are less well captured. The general pattern of these statistics is also captured, while the exact values for all degrees are not well captured by our model. The minimum geodesic distance, triad census, and core pattern of degrees are more relevant to our study due to our focus on general structural features rather than specific degree values.

### Gravity model of trade results

As discussed previously in the “ERGM” section, we found alignment between the ERGM and gravity model of trades results which the latter is commonly used in global trades network analysis (De Benedictis and Tajoli 2011; Fagiolo 2010; Dueñas and Fagiolo 2013). Our ERGM analysis uses gravity model results as the baseline results. The results for the gravity model analysis are provided in Table 9.

**Table 9:** Gravity Model estimates and standard errors alongside their significance on edge formation

Configuration	Estimate	Standard error	Significance
Intercept	− 2.985	0.144	***
GDP (Current US$) nodecov	− 0.054	0.019	**
GDP (Current US$) absdiff	0.301	0.025	***
Inflation, consumer prices nodecov	− 0.040	0.025	
Inflation, consumer prices absdiff	0.013	0.031	
GDP growth nodecov	0.015	0.020	
GDP growth absdiff	− 0.059	0.025	*
Is landlocked nodematch	− 0.274	0.040	***
Official language nodematch	− 0.188	0.071	**
Agriculture, forestry, and fishing, value added (% of GDP) nodecov	− 0.047	0.023	*
Agriculture, forestry, and fishing, value added (% of GDP) absdiff	0.078	0.028	**
Industry (including construction), value added (% of GDP) nodecov	0.091	0.023	***
Industry (including construction), value added (% of GDP) absdiff	− 0.146	0.028	***
Merchandise trade (% of GDP) nodecov	0.063	0.019	**
Merchandise trade (% of GDP) absdiff	− 0.016	0.026	
Net barter terms of trade index (2000 = 100) nodecov	0.084	0.018	***
Net barter terms of trade index (2000 = 100) absdiff	− 0.103	0.025	***
Foreign direct investment, net inflows nodecov	− 2.195	0.228	***
Foreign direct investment, net inflows absdiff	1.955	0.230	***

$$p<0.1$$^.^, $$p<0.05$$^*^, $$p<0.01$$^**^, $$p<0.001$$^***^

### Community detection

**Fig. 4:**
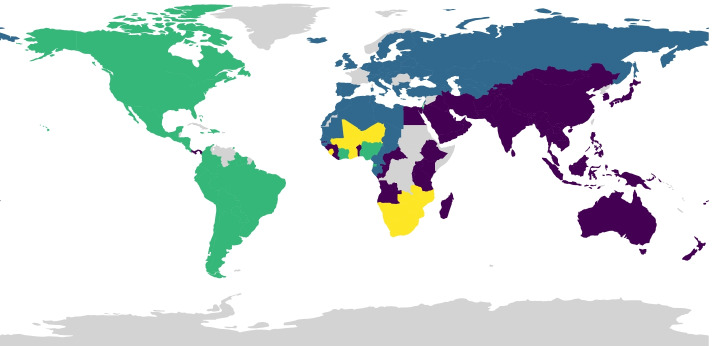
Community detection results depicted on the world map. The countries within each community are shown using the same color. Gray countries are not included in the analyses

Figure 4 depicts the community detection results on the weighted global trade network. The countries within the same community are shown with the same color on the world map. We found four communities:

- Green Community: It includes North America, South America, Côte d’Ivoire, and Nigeria.
- Blue Community: It contains European countries and several countries from North Africa.
- Purple Community: It includes most Asian countries, several countries from Africa, and Australia.
- Yellow Community: It includes African countries.

Observing the community detection results, it seems that countries with lower distances tend to form a single community; This conclusion was also seen through the ERGM results provided in Table 7. This also matches the gravity model of trades statement provided in “Gravity model of trade” section that suggests distance has a negative effect on trade exchanges volume. Analyzing the communities, we found Panama the only country not included in the North and South America continents’ community. Panama not belonging to the North and South America community can be explained through Panama’s geographic importance; Panama Canal connects Asian countries to the American countries and makes trade easier between these countries.

## Conclusion

In our study, we analyzed the global trade network from different perspectives. We constructed the essential trades network by applying the network backbone extraction methods. We used the ERGM methods on the global trade network; this investigation found various structural, economic, geographical, and political factors influencing the trade structure. The ERGM results showed structural features such as reciprocity and triangles impacting the trades. Moreover, nodal and edge features such as GDP, being landlocked, having diplomatic exchanges, and GDP percentages and distances between countries were also significant. We also studied the evolution of the trade network through multiple years. This analysis found factors influencing the formation and dissolution of trades, including structural features such as reciprocity, triangles, and non-structural features such as GDP, inflation, being landlocked, and official language affecting trades through time. Additionally, we applied community detection methods to analyze group structures in the global trade network. Furthermore, we studied the trade network using gravity models of trade which are frequently used in this context. We found our ERGM results consonant with the gravity model results. Our results could be insightful for policymakers helping them make better decisions. The ERGM results help policymakers understand better how the trades are formed in the network and act accordingly. STERGM can also help explain the effects of features through time on the formation of trades and, in particular, their dissolution. We hope that researchers put more effort and investment into using ERGMs or other network modeling methods to gain insights into complex networks in our social world, which some of them are as follows:

- How does Covid-19 affect the evolution of the global trade network?
- How do other statistical tests analyze the global trade network and its formation features?
- How can the global trade network be observed as a weighted network without applying backbone methods?
- How do other more complex structural features affect network formation in the global trade graph?

## Data Availability

All codes and datasets are provided in the following link: https://github.com/aminst/wits.

## Abbreviations

ERGM: Exponential Random Graph Model
DTERGM: Discrete Temporal Exponential Random Graph Model
STERGM: Seperable Temporal Exponential Random Graph Model

## Footnotes

1 https://wits.worldbank.org.

2 https://data.worldbank.org.

3 http://www.cepii.fr.

4 http://pardee.du.edu.
